# Solid-State Biology and Seed Longevity: A Mechanical Analysis of Glasses in Pea and Soybean Embryonic Axes

**DOI:** 10.3389/fpls.2019.00920

**Published:** 2019-07-16

**Authors:** Daniel Ballesteros, Christina Walters

**Affiliations:** National Laboratory for Genetic Resources Preservation, USDA-ARS, Fort Collins, CO, United States

**Keywords:** anhydrobiosis, aging, glass fragility, glass stability, longevity, mechanical analysis, seed, storage

## Abstract

The cytoplasm of anhydrobiotes (organisms that persist in the absence of water) solidifies during drying. Despite this stabilization, anhydrobiotes vary in how long they persist while dry. In this paper, we call upon concepts currently used to explain stability of amorphous solids (also known as glasses) in synthetic polymers, foods, and pharmaceuticals to the understand variation in longevity of biological systems. We use embryonic axes of pea (*Pisum sativum*) and soybean (*Glycine max*) seeds as test systems that have relatively long and short survival times, respectively, but similar geometries and water sorption behaviors. We used dynamic mechanical analysis to gain insights on structural and mobility properties that relate to stability of other organic systems with controlled composition. Changes of elastic and loss moduli, and the dissipation function, tan δ, in response to moisture and temperature were compared in pea and soybean axes containing less than 0.2 g H_2_O g^–1^ dry mass. The work shows high complexity of structure-regulated molecular mobility within dried seed matrices. As was previously observed for pea cotyledons, there were multiple relaxations of structural constraints to molecular movement, which demonstrate substantial localized, “fast” motions within solidified cytoplasm. There was detected variation in the coordination among long-range slow, diffusive and short-range fast, vibrational motions in glasses of pea compared to soybean, which suggest higher constraints to motion in pea and greater “fragility” in soybean. We are suggesting that differences in fragility contribute to variation of seed longevity. Indeed, fragility and coordination of short and long range motions are linked to stability and physical aging of synthetic polymers.

## Introduction

Long-term survival of dry germplasm living in soils or seed banks (i.e., anhydrobiotes) has serious economic and ecological implications, worth billions of dollars annually to agriculture and conservation groups (Li and Pritchard, 2009). Anhydrobiotes are metabolically quiescent (Vertucci and Farrant, 1995; Rebecchi et al., 2007; Sajeev et al., 2019), but major changes occur with time in these dry systems, affecting eventual recovery once hydrated. An organism may be induced to grow by a desiccation-rehydration cycle (Leubner-Metzger, 2005; Bazin et al., 2011), or may age and eventually lose viability (Walters et al., 2005a, 2010; Sano et al., 2016). Much of the changed physiology depends on the extent and duration of drying. Yet, because the organism is quiescent, we have a poor understanding of the biochemical or biophysical changes associated with gained or lost function while the organism is dry.

Dry organisms are fundamentally different than hydrated ones because their cytoplasm is solidified as opposed to fluid. As a reminder, solids are distinguished from fluids because they maintain shape (i.e., structure), at least within experimentally tractable time-scales. Solids form by compressing a fluid mixture of molecules closely together so that neighboring molecules entrap each other and create structure by restricting movement. Amorphous, as opposed to crystalline, solids lack regularity in the packing density (also referred to as unoccupied volume or pore space). Temperature effects on solidification receive strong theoretical support (e.g., Angell, 1995; Walters, 2004; Wowk, 2010; Roos et al., 2016), and a core property of materials is their solidification temperature (Tm for crystals and Tg for amorphous solids). Tg increases with the volume occupied by a molecule, which is mostly affected by the rigidity of the polymer backbone (Kunal et al., 2008; Stukalin et al., 2010).

Glass fragility (*sensu* Angell, 1995) is another core property of amorphous solids (Angell et al., 2000). A fragile glass has considerable pore space within the matrix as a consequence of inflexible, bulky molecules that do not compress well. A fragile glass changes abruptly from solid ↔ fluid over a narrow temperature range, exhibiting a discontinuous Arrhenius plot for viscosity-related events at Tg. In contrast, viscosity-related events in a strong glass follow Arrhenius kinetics because changes occur over a broad temperature range (see Ballesteros et al., 2018 for more detail). Fragility purportedly arises from space filling properties of molecules that relate to coordinated folding of polymer backbone and sidechains in response to lower temperature or anti-plasticization (Dudowicz et al., 2008; Kunal et al., 2008; Stukalin et al., 2010). In essence, strong glasses are comprised of flexible molecules in which polymer backbones and side chains exhibit similar folding patterns in response to reduced temperature or lost diluents or plasticizers.

Molecular motions within a solid are constrained but not absent. Depending on intermolecular distances, molecular flexibility and co-ordinated short and long range movements, molecules can slide past each other or realign within a confined neighborhood. Motions are classified as slow/long-range/diffusive or fast/short-range/rotational or vibrational (Yoshioka and Aso, 2007; Bhattacharya and Suryanarayanan, 2009). Diffusive motion is facilitated by loosening of rigid structure which opens unoccupied volume in a process coined “α relaxations.” Fast, short range motions, like ligand rotations or vibrations, still occur in solids and exhibit different temperature sensitivity than diffusive motion. In a solid, temperature increases may ease volume constraints and facilitate fast, localized motions known as β or γ relaxations (Angell et al., 2000). These fast, short range motions are increasingly characterized in materials of known composition because they have important effects on the stability of the solid matrix. They are correlated with α relaxations, solvent penetration, gas diffusion, physical aging, and brittleness leading to microstructural changes of foods and polymers (Menard, 1999; Perez et al., 1999; Khare et al., 2000; Roudaut et al., 2004; Zhang et al., 2005; Poirier-Brulez et al., 2006).

Additives or diluents, change core properties of the solid by affecting rigidity of molecular backbones and side chains or the distances among neighboring molecules (Smith, 2003; Zhang and Han, 2006; Stukalin et al., 2010; Choi et al., 2011; Silalai and Roos, 2011). Anti-plasticizers can make the matrix denser or “tougher” and plasticizers, like water, can reduce rigidity and increase intermolecular spacing (Ball, 2008; Buitink and Leprince, 2008; Sokolov et al., 2008; Doster, 2010; Silalai and Roos, 2011). It would seem, that in the context of the complex mixture that is cytoplasm, there may be limitless options to control structure and the response of structure to temperature and moisture. Perhaps this contributes to the role of LEA-like proteins that are linked to stability of dry biological systems (Mouillon et al., 2008; Chatelain et al., 2012). Heterogeneity in polymer or additive concentration within cytoplasm can profoundly affect localized response (e.g., Leubner-Metzger, 2005; Choi et al., 2011; Hyman and Simons, 2012).

The complex composition and thermal history of biological glasses make these materials not very accessible to investigation using spectroscopic relaxation techniques more commonly used to study polymer behavior. Sample preparation, time scale of measurement and inability to distinguish α and β components also confound interpretation. Mostly, the glass ↔ fluid transition is detected using differential scanning calorimetry (DSC), and Tg signals (changed heat capacity, ΔCp) are small and occur over a broad temperature range. The studies indicate that plasticization curves (i.e., Tg response to changes in moisture) are similar among cytoplasm from diverse organisms ranging in desiccation tolerance (Buitink and Leprince, 2008; Walters et al., 2010). A few studies have attempted to characterize molecular mobility or structure within dried cytoplasm (Bruni et al., 1989; Buitink et al., 2000; Walters, 2004; Ballesteros and Walters, 2011). These techniques give much greater sensitivity than simple DSC scanning, but they are laborious and subtle effects are difficult to perceive. We have reasoned that better understanding of dry biological glasses will come from more detailed studies of solid properties in cytoplasm and how these change with time.

Glasses are dynamic structures and so we expect properties to change with time as molecules move in a process known as physical aging (Struik, 1977). There is an inextricable, though still unclear, relationship between glass fragility, β relaxation and physical aging of the glass (McKenna and Simon, 2002; Micoulaut, 2016). Local, fast β motions precede, and even instigate, longer-range slower α motions (Angell et al., 2000; Priestley et al., 2005; Yu et al., 2013). Fragile glasses physically age faster because molecules are packed less efficiently (Angell et al., 2000; Yu et al., 2013; Micoulaut, 2016). Physical aging is often accompanied by chemical changes as molecules come into increasingly closer proximity (Yoshioka and Aso, 2007; Bhattacharya and Suryanarayanan, 2009). These chemical changes (e.g., carbonylation and crosslinking) as well as continued compression of molecules lead to greater brittleness in an aged glass. Lost functionality ensues, and in polymers, it remains unclear whether the physical or chemical aging is causative.

Many of the properties of glasses refer to mechanical stresses or responses to mechanical stresses (e.g., compression, rigidity, and toughness) and, indeed, mechanical characterization of glasses provides a highly sensitive method to evaluate material properties and predict future changes in performance, especially of the macroscale (Menard, 1999). Instrumentation to evaluate mechanical properties in polymers have been adapted to obtain precise and repeatable measures in dry biological samples, such as seeds (Walters et al., 2010; Ballesteros and Walters, 2011), and moss tissues (Fernández-Marín et al., 2013). Mechanical analysis measures the deformation of a material in response to an applied force. The stiffness of the material is expressed by the storage modulus (E′), which represents recoverable deformation (like a spring), where energy is conserved. The tendency of molecules within the material to flow (or lose energy) is expressed by the loss modulus (E^″^). By using an oscillating force, dynamic mechanical analysis (DMA) measures the ratio of conserved versus lost energy, tan δ (also called the energy damping or dissipation factor) using the phase angle between the force and deformation waves (Menard, 1999). In an ideal spring, tan δ = 0 because no energy is lost. The value of tan δ increases as the material becomes more fluid because energy is lost by molecular rearrangements and internal friction.

The purpose of this paper is to advance understanding of solidified cytoplasm using DMA approaches. Pea (*Pisum sativum*) and soybean (*Glycine max*) seeds are used as experimental systems because their similar geometries simplify DMA interpretation and their differences in longevity and response to moisture provide context for observed changes in properties in response to moisture or temperature (Vertucci and Roos, 1990; Walters et al., 2005b; Fleming et al., 2017, 2018). Here, our analyses focus on the embryonic axis and relationships between α and β relaxations. Our goal is to identify mechanical properties that distinguish solids within pea and soybean tissues and to detect discontinuities in these properties in response to moisture that may explain discontinuities in physiological aging responses to moisture.

## Materials and Methods

### Plant Material and Sample Preparation

Seeds of soybean (*Glycine max* ‘Williams 1982’) and pea (*Pisum sativum* ‘Alaska’) were purchased from Missouri Seed Foundation (Columbia, Missouri, United States) and De Bruyn Seed Co., Inc. (Zeeland, Michigan, United States), respectively. The seeds used for DMA were from 2010 harvests and for seed longevity were from a range of harvests dating from 1989 to present. The effect of RH on aging rate used seeds from 1991, 1992, and 1994 harvests. Seeds were cleaned by the vendor, shipped to Fort Collins, CO within 3 months of harvest and then stored at 5°C and 30–50% RH until used. Under these conditions, seed viability for soybean and pea begins to decline after about 15 and 30 years, respectively (Fleming et al., 2017, 2018). No changes were observed in DMA parameters in either species over the 2 years time frame of the experiments, allowing us to pool measurements.

For DMA, seed coats were removed from dry seeds and the exposed embryonic axis was excised using a dissecting needle. Embryonic axes were selected for similar size and shape to reduce variation in the DMA signals. Axis dimensions in both species ranged from 5 to 6 mm long and 1.5 to 2 mm wide.

Water content of whole seeds (aging studies) and embryonic axes (DMA) was adjusted between 0.01 and 0.22 g H_2_O g^–1^ dw (g g^–1^) by placing samples at relative humidity (RH) between 0 and 92%. At room temperature, RH was controlled using saturated salt solutions to give nine RH levels ∼1, 5.5, 13, 25, 33, 43, 50, 62, and 75% (Vertucci and Leopold, 1987) and 85 and 92% RH treatments were added for 35°C treatment in whole seed aging studies. In order to express all water contents in terms of the embryonic axes, water contents measured for whole seed during aging were converted to water contents of axes using water sorption isotherms as described previously (Vertucci and Leopold, 1987).

For DMA studies, fresh mass was measured before axes were loaded on to the DMA and after DMA measurements. Changes in axis water content during heating were corrected by monitoring mass at 20°C intervals above 50°C (data not presented, Ballesteros and Walters, 2011). Samples containing more than 0.15 g H_2_O g^–1^ dw began to dry at 50°C and samples containing more than 0.03 g H_2_O g^–1^ dm began to dry at 90°C. We calculated water loss rates for each species, water content and temperature range and used these relationships to adjust water content at which relaxation events were observed. Dry mass was measured by holding axes and whole seeds at 95°C for 36 h and 3 days, respectively. Water content is calculated from the difference between fresh and dry weight and is expressed on a dry mass basis as g H_2_O g^–1^ dw.

### Seed Aging

Experiments to measure the effect of water content on aging rates of whole seeds of pea and soybean were conducted at 35°C using three separate cohorts of seeds harvested in the early 1990s (Vertucci and Roos, 1990). Seeds were stored over the saturated salt solutions and monitored for viability at weekly, monthly or quarterly intervals, depending on the expected aging rates. Data describing loss of viability with time were pooled for each moisture treatment and fit to an Avrami equation to calculate P50 (Walters et al., 2005a,b).

### Dynamic Mechanical Analysis (DMA)

Visco-elastic properties of pea and soybean embryonic axes were analyzed using a DMA-7e analyzer (Perkin-Elmer, United States) over the temperature range of −120 to 130°C (up to 200°C for axes containing 0.02 g H_2_O g^–1^ dm). DMA tests were conducted in the parallel plate compression mode using a 1 mm probe. Axes were placed on the base plate in a stable position to ensure contact of both the base plate and probe in the middle of the axis (Figure 2). Static force was set at 200 mN and dynamic force was set at 165 mN, delivered at a frequency of 1 Hz during warming (Ballesteros and Walters, 2011). Samples were cooled to −120°C at a rate of 10°C min^–1^, held at −120°C for 2 min, and then heated to 130°C at a rate of 3°C min^–1^. Liquid nitrogen was used as the coolant and helium as the purge gas (20 mL min^–1^). The instrument returned the displacement (deformation) of the sample at the site of the probe as well as the phase lag between stress and strain waves. With the force and frequency settings, displacements amplitudes usually ranged from 0.5 to 5 μm and phase angles were usually between 2 and 20 rad, depending on the water content of the sample and the temperature during the scan.

Stress-strain relationships during force oscillations were used to calculate storage (E′) and loss (E^″^) modulii and tan δ (E^″^/E′) using the manufacturer’s software. Stress is measured as force ÷ area (i.e., pressure) and strain is measured as displacement ÷ original size (dimensionless) and so both modulii are measured in pressure units (pascals). The relative loss of energy during the applied force as a result of viscous flow is known as damping and is indicated by the loss or dissipation factor, called tan δ, where tan δ = E^″^/E′(Menard, 1999). Tan δ values tend to peak at Tg and then decline when motion is dominated by viscous flow. Variation in geometry has large effects on E′ and E^″^ but is corrected in tan δ measurements, making tan δ a particularly useful parameter to summarize modulus changes while controlling for sources of experimental variability. In our experiments, relaxation events were identified primarily through peak or stepwise changes in tan δ as a function of temperature, and were confirmed by discontinuities in the relationships between temperature and either storage or loss moduli. While visualizing scans for assessment of relaxation properties, we used Perkin Elmer Pyris software to smooth scans in the vicinity of very small relaxations, e.g., γ. Onset temperatures were calculated using the Pyris onset calculation tool. Peaks were calculated using the Pyris peak tool, when possible, or from the intersection of the lines drawn after the onset but before the peak and after the peak. The gamma relaxation was large enough to reliably detect peaks; but we could not reliably detect an onset using our methods. Computed values are provided for each sample-treatment combination.

Dynamic mechanical analysis scans were collected on at least 4 different axes for each moisture-species treatment. *T*-test or ANOVA were used to compare measured properties within and among species and water content ranges. After ANOVA, *post hoc* analyses (Tukey test) were performed to compare averages between groups. The significance of plasticization effects was tested by linear regression using functions available in Excel software. Pearson’s correlation coefficients were calculated to quantify the strength of association between temperature and water content of the diverse relaxations.

## Results

**FIGURE 1:**
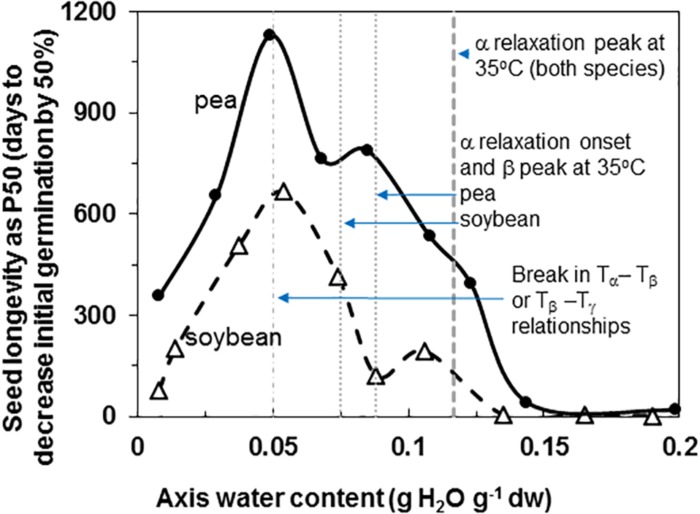
Longevity of soybean (open triangles) and pea (solid circles) seeds stored at 35°C and RH ranging from 1 to 92%. The water contents indicated are for embryonic axes treated at the specific RH/temperature combination, which was determined using water sorption isotherms similar to those given by Vertucci and Leopold (1987). The P50 values (time for viability to decrease to 50% was calculated from deterioration time courses (not shown) that were fitted to an Avrami equation (Walters et al., 2005a,b). The vertical lines indicate key properties of the solid at 35°C that were characterized by DMA measurements: water content for the peak and onset of α relaxations (Figure 6) and breaks in the pattern of separation of different relaxation events (Figure 7).

Pea and soybean seeds age with time and the rate is dependent on temperature (data for 35°C provided) and water content (Figure 1). Longevity is expressed as time for 50% of seeds to die, P50, and ranged from a low of 2 and 19 days to a maximum of 666 and 1129 days for soybean and pea, respectively. Maximum longevity was observed at 33% RH (0.04 g g^–1^) for soybean and 15% RH (0.05 g g^–1^) for pea seeds stored at 35°C. Pea maintained at least a 2-fold higher P50 at supra-optimum water contents, which is consistent with most comparisons between the species (Priestley, 1986; Walters et al., 2005b; Fleming et al., 2017, 2018; Royal Botanic Gardens Kew, 2019). At sub-optimal water contents, P50 values for pea and soybean seemed less differentiated.

**FIGURE 2:**
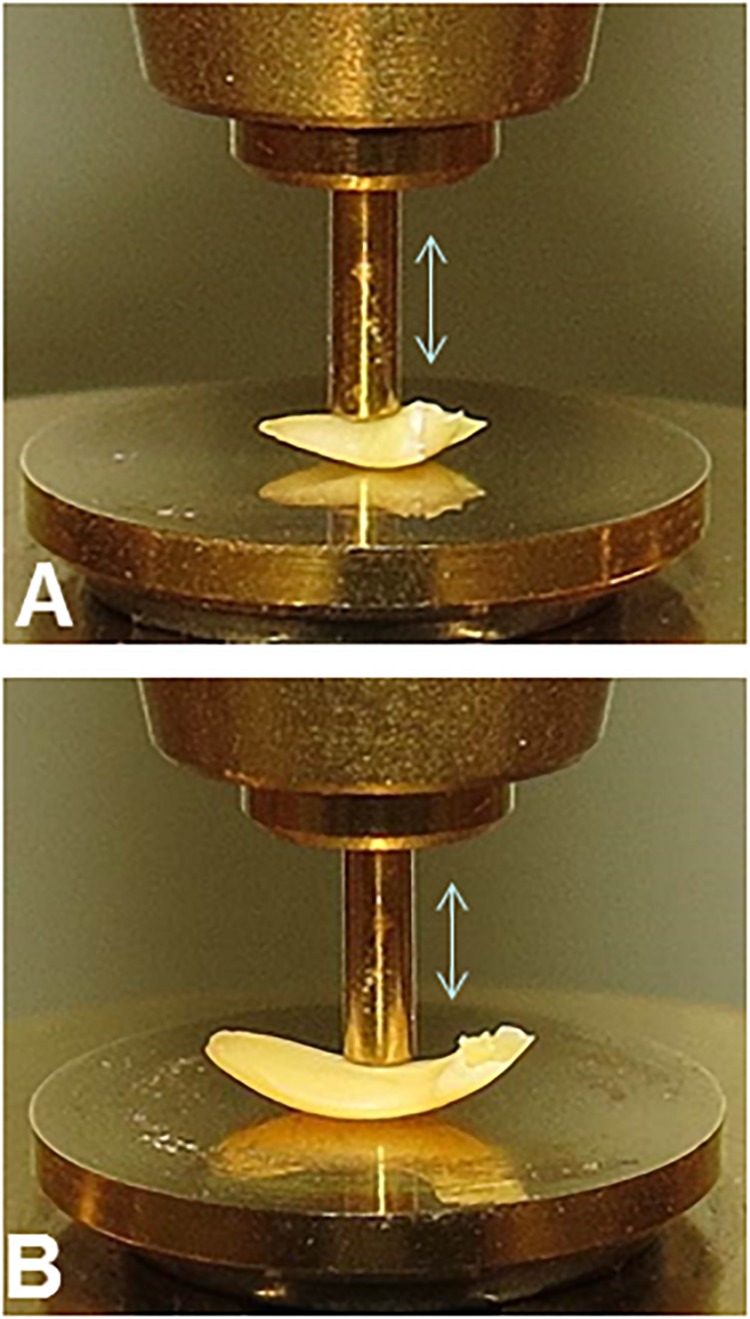
Experimental set-up for DMA experiments on pea **(A)** and soybean **(B)** embryonic axes. The 1 mm diameter probe was placed on the convex surface of axes between the plumule and the radicle to deliver force evenly across surface.

The similar geometries of embryonic axes excised from pea and soybean seed make them more amenable to comparison using mechanical analyses (Figure 2). Even still, variation in geometry within and among samples is a major source of experimental variation in DMA measurements confounding comparisons between pea and soybean except for the dissipation factor (tan δ), which corrects for geometry.

**FIGURE 3:**
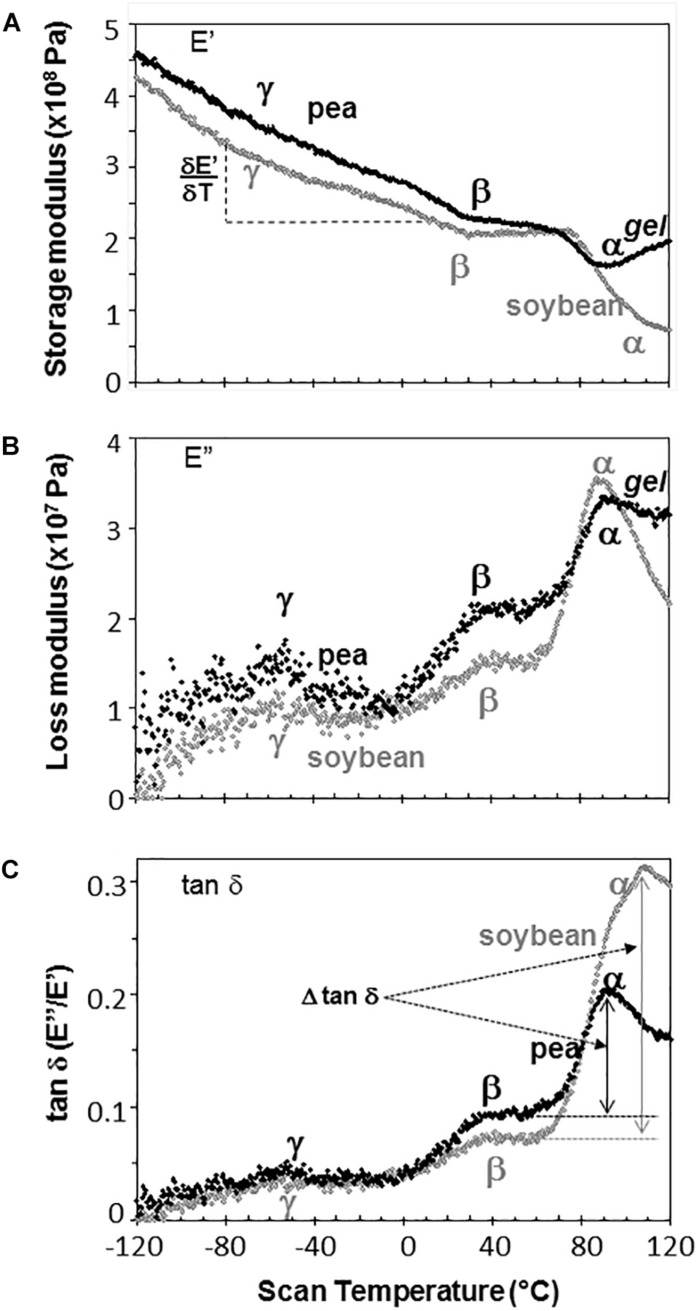
The relationship between temperature and storage modulus **(A)**, loss modulus **(B)**, and tan δ **(C)** functions in pea (black symbols) and soybean (gray symbols) embryonic axes containing 0.05 g H_2_O g^–1^ dry mass. Relaxation events are labeled as α, β, and γ, according to convention in mechanical analyses. The slope of the storage modulus with temperature $\left(\frac{\partial E^{\prime }}{\partial T}\right)$ was calculated from the points between –80 and +20°C and the relationship of this slope to changes in moisture is summarized in Table 1. The size of α relaxation (Δ tan δ) was determined from the baseline to the peak of the tan δ curve, as indicated and changes in this parameter with moisture are summarized in Figure 8. Scans are representative of replicate treatments.

Representative DMA scans of pea and soybean embryonic axes containing 0.05 g H_2_O g^–1^ dw illustrate how decreasing temperature results in an overall increase in E′ (i.e., the sample becomes stiffer) (Figure 3A) and decrease in E^″^ (i.e., sample becomes less fluid and less susceptible to permanent deformation) (Figure 3B). There was considerable variation in E′ and E^″^ values for each species-water content-temperature combination (scans of replicate treatments not shown). For example, at 0°C, E′ for pea and soybean axes containing 0.05 g H_2_O g^–1^ dw was 215 ± 66 MPa and 237 ± 20 MPa, respectively (*T*-test, *P* > 0.05). This variation most likely results from geometry differences among samples or differences in the placement or contact of the probe with the sample. Values for E^″^ were highly scattered below −40°C (Figure 3B), which indicates that the combination of amplitude and phase angle were not always optimized under dry or very low temperature conditions (modifying force parameters for these specific conditions should alleviate some of the “noise” in future studies). Tan δ calculations normalize differences in geometry and allowed us to detect significant differences between pea and soybean axes [for example, axes containing 0.05 g g^–1^ have average tan δ values 0.074 ± 0.012 and 0.052 ± 0.011 at 5°C, respectively (*T*-test, *P* < 0.05)] (Figure 3C).

The slope of the E′ relationship $\left(\frac{\partial E^{\prime }}{\partial T}\right)$ was calculated between −80 and 20°C to describe the sensitivity of the modulus to temperature change (Figure 3A). For axes containing 0.05 g H_2_O g^–1^ dw, the average $\frac{\partial E^{\prime }}{\partial T}$ was −1.09 ± 0.41 and −1.25 ± 0.22 MPa°C^–1^ across 5 replicates of pea and soybean axes, respectively, indicating similar responses of the two tissues to temperature at this water content (*T*-test, *P* > 0.05). Similar calculations of the slope of E^″^ or tan δ relationships with temperature revealed no significant differences between pea and soybean axes containing 0.05 g H_2_O g^–1^ dm. Above 80°C, E′ decreased in soybean axes, but increased in pea axes, the latter reflecting stiffening of the material, probably due to drying or starch gelation (indicated by ‘gel’ in Figure 3).

Step-down or step-up discontinuities in storage and loss modulii, respectively, were observed in pea and soybean axes containing 0.05 g H_2_O g^–1^ dw at about 90°C, 40°C, and −60°C (Figure 3). These discontinuities reflect molecular relaxations and are most clearly discerned in the tan δ function (Figure 3C). The highest temperature relaxation, α, indicates the transition of the material from solid ↔ fluid and so reflects Tg. This relaxation was larger (indicated by length of arrows at the tan δ peak, Δ tan δ) than the β and γ relaxations. Temperatures for α relaxations were about 10°C lower in pea compared to soybean axes containing 0.05 g H_2_O g^–1^ dw. A smoothing tool provided in Perkin Elmer software made it easier to discern γ relaxations through the scatter of points.

**FIGURE 4:**
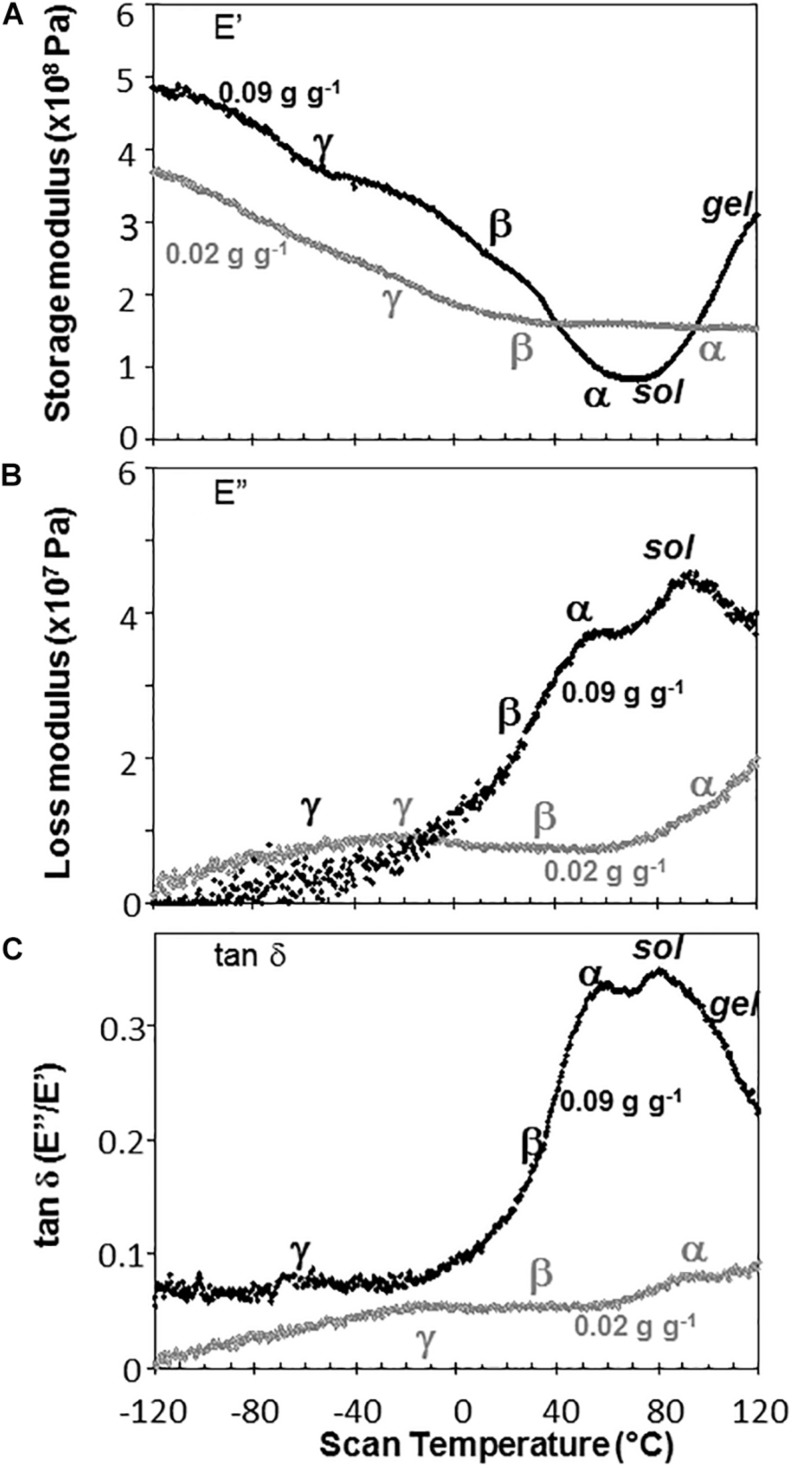
The relationship between temperature and storage modulus **(A)**, loss modulus **(B)**, and tan δ **(C)** functions in pea embryonic axes containing 0.09 (black symbols) and 0.02 (gray symbols) g H_2_O g^–1^ dry mass. Relaxation events are as labeled in Figure 3. Additional high temperature transitions, typical of starchy materials, are also labeled.

**FIGURE 5:**
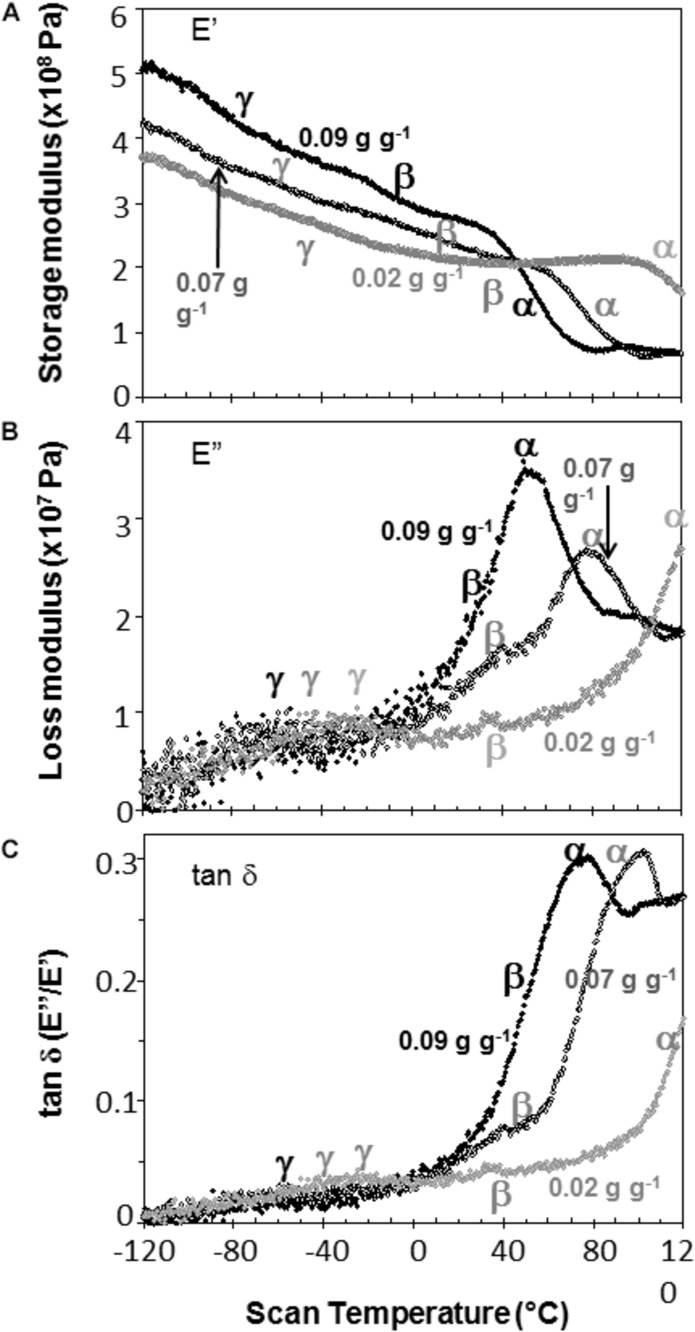
The relationship between temperature and storage modulus **(A)**, loss modulus **(B)**, and tan δ **(C)** functions in soybean embryonic axes containing 0.09 (black symbols), 0.07 (black, open symbols), and 0.02 (gray symbols) g H_2_O g^–1^ dry mass. Relaxation events are as labeled in Figure 3.

Water content had strong effects on the characteristics of DMA scans in pea and soybean axes [representative scans provided in Figures 4 (pea) and 5 (soybean)], but these were difficult to quantify by E′, E^″^ or tan δ values at specific water content-temperature combinations. For example, E′ appeared lower in dry compared to wetter pea axes (Figure 4A); however, there was too much variation in E′ measurements to discern a statistically significant difference (for example, *E*′ at 0°C was 246 ± 117, 215 ± 66, and 184 ± 76 MPa, in pea axes containing 0.09, 0.05, and 0.02 g H_2_O g^–1^ dw, respectively. Average E′ at 0°C was 257 ± 36, 237 ± 20, and 233 ± 46 for soybean axes at similar water contents). Alternatively, we correlated the slope of the temperature scan (i.e., $\frac{\partial E^{\prime }}{\partial T}$, Figure 3A) with water content and found a significant relationship for pea but not for soybean axes [Table 1; *P* < 0.05 (pea), *P* > 0.05 (soybean)]. Data for pea cotyledons (Ballesteros and Walters, 2011) are added to Table 1 for comparison and demonstrate a significant relationship with water content (*P* < 0.05) that is similar to pea axes (*P* > 0.05). Indeed, the mechanical characteristics between −120 and 20°C hardly changed in very dry pea axes (scans are nearly flat with few discontinuities), while steeper scans and numerous discontinuities are visible in more hydrated pea axes (Figure 4). The parallel changes of $\frac{\partial E^{\prime }}{\partial T}$ with water content in pea axes and cotyledons (Table 1), with the intercept approaching 0 for pea cotyledons, implies that this material becomes nearly inert when it is completely dry. In contrast, mechanical properties in soybean axes (Figure 5) indicate some level of flexibility, even under extremely dry conditions, which explains the relatively low slope, but high absolute value of intercept in $\frac{\partial E^{\prime }}{\partial T}$ versus moisture correlations (Table 1).

**TABLE 1:** Regression parameters for relationships between water content (ranging from 0.01 to 0.12 g H_2_O g^–1^ dw) and the slope of E′ versus temperature $\left(\frac{\partial E^{\prime }}{\partial T}\right)$ between −80 and +20°C (DMA scans in Figures 3–5 are representative).

**Species and**	**Slope (MPa**	**Intercept**	**Pearson**	**Number of**
**tissue type**	**g dm°C^–1^**	**(MPa ⋅ °C^–1^**	**correlation**	**samples**
	**g^–1^ H_2_O)**	**for wc = 0 g**	**coefficient**	
		**H_2_O g^–1^ dm)**		
Pea axes	−13.6±3.0^a^	−0.77±0.20^a^	0.32	47
Soybean axes	−1.9±2.3^b,*ns*^	−1.29±0.15	0.02	35
Pea cotyledons	−9.7±1.9^a^	−0.37±0.19^b^	0.34	50

*^*ns*^regression is not significant at *P* > 0.05. The analysis for pea cotyledons is also provided using data from Ballesteros and Walters (2011). Regressions for pea axes and cotyledons were significant (*P* < 0.05), but those for soybean axes were not significant. Slopes marked with different superscripts were significantly different (*P* < 0.05). Intercepts having different superscripts were significantly different (*P* < 0.05) for lines with the same slopes (i.e., the lines are parallel).*

**FIGURE 6:**
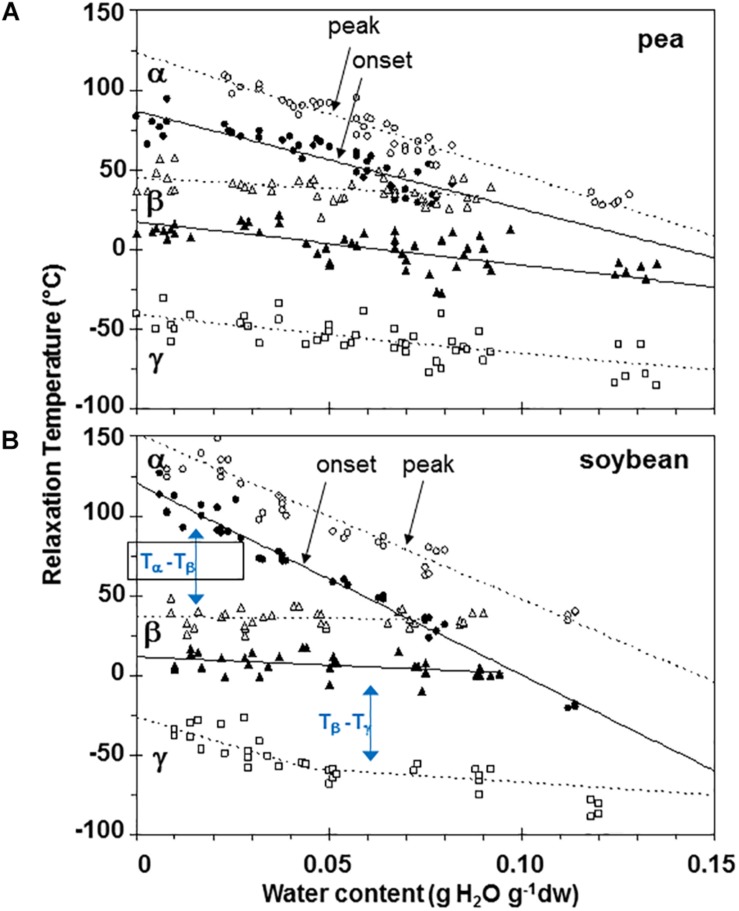
Plasticization curves for glasses of pea **(A)** and soybean **(B)** embryonic axes showing the relationships between water content and temperature of α (circles), β (triangles), and γ (squares) relaxations. Data are provided for the onset of the relaxation (filled symbols) and peak (open symbols). Lines are the calculated regression relationships (Table 2). Two lines were calculated for the γ relaxation data for soybean using data ≥ and ≤0.05 g H_2_O g^–1^ dry mass.

Moisture effects on Tg (i.e., plasticization) are also evident from the size and temperature of discontinuities, step functions or peaks in the DMA scans (Figures 3–5, summarized in Figure 6). The α relaxation in pea and soybean axes containing 0.02 g H_2_O g^–1^ dm occurred near 90°C and is small for pea (Figures 3, 4). Heating pea axes above 120°C, resulted in large fluctuations in tan δ, reminiscent of decomposition through starch gelation, protein denaturation, sugar melting and volatilization (data not shown), and axes were charred by this treatment. Increasing moisture led to decreasing temperatures of α relaxations (Pearson coefficient analysis, *P* < 0.05) (Table 1). Moisture had less, but still significant, effects on the temperatures of β and γ relaxations (Pearson coefficient analysis, *P* < 0.05) (Table 1). We analyzed the moisture versus temperature of γ relaxation in soybean axes as two lines to account for an apparent steeper relationship with temperature at water contents ≤0.05 g g^–1^. The intercept of the plasticization effects describe α (i.e., Tg), β and γ relaxation of completely dry materials, an often used parameter in material sciences (Table 2). Pea axes had lower α and γ relaxation temperatures and higher β relaxation temperatures than soybean axes. The properties of pea axes and cotyledons could also be distinguished. The slopes for water content effects on α and β relaxations differed in both pea and soybean (Table 2), leading the two lines to intersect at 0.078, 0.096, and 0.142 g g^–1^ for soybean axes, pea axes and pea cotyledons, respectively. This can be visualized in DMA scans as merged α + β relaxations near 40–60°C in axes containing 0.09 g H_2_O g^–1^ dm (Figures 4, 5). Assuming continuation of the linear α relaxation relationship with water content, the extrapolated α + β relaxation intersected the γ relaxation at −116°C and 0.31 g H_2_O g^–1^ dm (∼92% RH at 22°C) and for pea axes, at −83°C and 0.19 g H_2_O g^–1^ dm (∼85% RH at 22°C) for soybean axes, and at −115°C and 0.46 g H_2_O g^–1^ dm (RH > 96%) for pea cotyledons (data not shown).

**TABLE 2:** Regression parameters for relationships between water content and temperature of α, β, and γ relaxations events (onset and peaks).

**Regression parameter**	**Species and tissue type**	**α relaxation (onset)**	**β relaxation (peak)**	**β relaxation (onset)**	**γ relaxation (peak)**
Slope (°C g dw g^–1^ H_2_O)	Pea axes	−669±36^b^	−259±38^d^	−252±32^d^	−244±27^d^
	Soybean axes	−1029±50^a^	−21±35^*ns*^	−108±41^e^	−731±94^b,1^
					−161±54^e,2^
	Pea cotyledons	−452±28^c^	3±28^*ns*^	−131±28^e^	−169±29^e^
Intercept	Pea axes	88±2	49±3	16±2	−42±2
(°C for wc = 0 g H_2_O g^–1^ dw)	Soybean axes	115±2	37±2	12±2	−27±3
					−52±5
	Pea cotyledons	95±2	30±2	7±2	−37±3

*^1^refers to the relationship at water content ≤0.05; ^2^refers to the relationship at water content ≥0.05; ^*ns*^regression is not significant at *P* > 0.05. Data are from Figure 6. Regressions were significant (*P* < 0.05) except for moisture effects on the temperature of the β relaxation peak. Slopes marked with different superscripts were significantly different (*P* < 0.05). Analysis for pea cotyledons are provided for comparative purpose and come from Ballesteros and Walters (2011). The γ relaxations in soybean axes were analyzed for water contents above and below 0.05 g H_2_O g^–1^ dw.*

**FIGURE 7:**
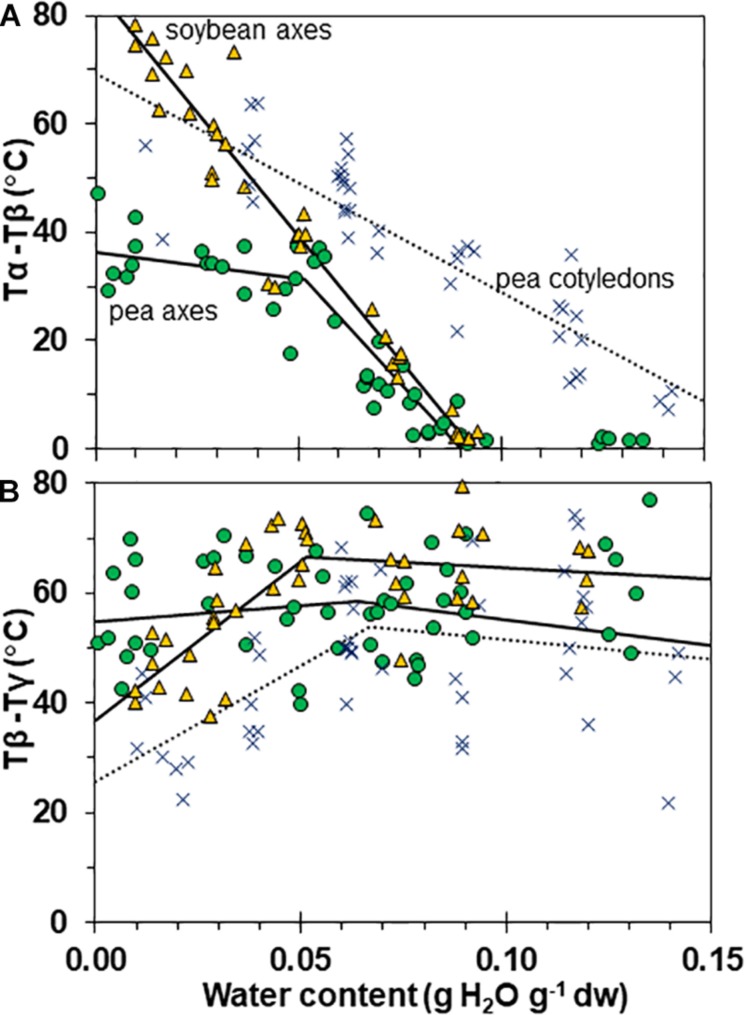
Temperature differences separating α and β **(A)** and β and γ **(B)** relaxation events in pea (solid circles) and soybean (open triangles) embryonic axes. Data for pea cotyledons (Ballesteros and Walters, 2011; blue Xs) are provided for comparative purposes. In **(A)**, the temperature for the onset of α relaxation is subtracted from the peak of the β relaxation and in **(B)**, the temperature for the onset of the β relaxation is subtracted from the peak of the γ relaxation. The lines represent regression relationships calculated for tissues having water contents ≥ and ≤0.05 g H_2_O g^–1^ dw. In **(A)**, relationships were significant (*P* < 0.05) for T_α_ – T_β_ in soybean axes (all wc), pea cotyledons (all wc) and pea axes (wc ≥ 0.05 g H_2_O g^–1^ dw). The slopes for pea and soybean axes were similar (*P* > 0.05) and significantly greater (*P* < 0.05) than pea cotyledons. The x intercepts were calculated as 0.09 g H_2_O g^–1^ dw for pea and soybean axes and 0.17 g H_2_O g^–1^ dry mass for pea cotyledons. In **(B)**, relationships were significant (*P* < 0.05) for T_β_ – T_γ_ in only soybean axes and pea cotyledons at water contents ≤0.05 g H_2_O g^–1^ dw.

Interactions between α, β, and γ relaxations reveals finer detail of solid properties and may provide insight about physiological behavior. The temperature differences between α (onset) and β (peak) as well as β (onset) and γ (peak) relaxations were calculated for individual samples (Figures 7A,B, respectively). The overlap of α and β relaxations are indicated by a 0°C difference at water contents near 0.09 g g^–1^ for axes of both species. Distinction between these relaxations increases steadily with drying in soybean axes, but only to about 0.05 g H_2_O g^–1^ dw in pea axes after which the difference in temperature between these relaxations is more oblique (Figure 6A). An analogous creep of γ toward β relaxations is noted in soybean seeds at water contents less than 0.05 g H_2_O g^–1^ dw (Figure 7B).

**FIGURE 8:**
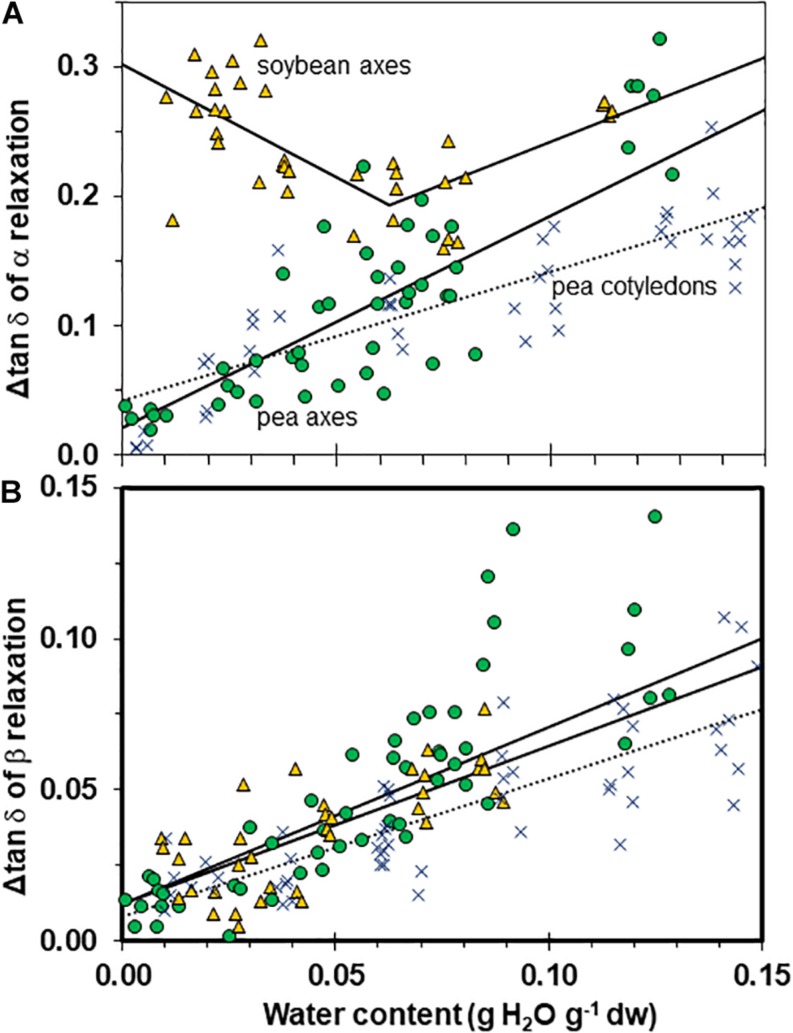
The size of α **(A)** and β **(B)** relaxation signals in pea (solid circles) and soybean (open triangles) embryonic axes. Data for pea cotyledons (Ballesteros and Walters, 2011; blue Xs) are provided for comparative purposes. Signal size was calculated from Δ tan δ functions as indicated in Figure 3C. The lines represent regression relationships calculated for all water contents except for soybean axes in **(A)**, where regressions were calculated separately for water contents ≥ and ≤0.05 g H_2_O g^–1^ dw. All regressions were significant (*P* > 0.05). Slopes for the size of α relaxation were similar in pea and soybean embryonic axes at water contents ≥0.05 g H_2_O g^–1^ dw (*P* > 0.05) and for β relaxation were similar for all tissues.

We characterized the size of relaxations through tan δ signals to estimate the number of molecules participating in the transition (Figure 8A). Transition size decreased with increased drying in a similar way for axes of both pea and soybean when water content was >0.05 g g^–1^ (i.e., slopes for axes were not significantly different, *P* > 0.05). However, the relationships for pea axes and cotyledons were significantly different (*P* < 0.05) (Figure 8A). In soybean axes dried to ≤0.06 g H_2_O g^–1^ dw, Δ tan δ appeared to increase with decreasing moisture. Alternatively, one could interpret that Δ tan δ does not vary with moisture in soybean axes. More data are needed to elucidate the moisture effects on soybean. Despite difficulties interpreting the pattern of moisture effects on soybean axes, it is clear that α relaxations are larger in this species compared to either tissue of pea (intercepts for regressions were also significantly different, *P* < 0.05).

The β signal was small in both species and tissue types, and decreased shallowly as water content approached 0 (Figure 8B, *P* < 0.05, *r*^2^ = 0.54, 0.52, 0.71 for pea axes, soybean axes, pea cotyledons (not shown), respectively. All tissues appeared to follow a similar relationship with water (i.e., slopes were not significantly different, *P* > 0.05).

## Discussion

In this paper, we examined mechanical properties of solidified cytoplasm in pea and soybean embryonic axes over a range of temperatures and water contents relevant to seed preservation. A major relaxation (α) reflects long-range (slow) diffusive motion that marks the transition of the material from solid ↔ fluid at temperatures between 25 and 100°C in axes dried at <50% RH and room temperature. We also confirm that the solid (or glass) allows short-range (fast) molecular movements above 40 and −30°C, identified by the presence of β and γ relaxations (Ballesteros and Walters, 2011). Plasticization relationships describe temperature-water content combinations at which these relaxations are observed. Those measured using DMA (Figure 5) provide greater insight of motion within a solid compared to more commonly used calorimetric techniques that measure Tg (Buitink and Leprince, 2008). However, plasticization alone does not reveal sufficient differences among glasses in different species of seeds to explain different physiology.

The α relaxation (comparable to Tg) is most frequently used to confirm that the material has, in fact, solidified and to crudely differentiate the properties of materials differing in composition. Here, we show that cytoplasm of embryonic axes containing less than 0.1 g H_2_O g^–1^ dm is unequivocally solid at temperatures ≤50°C (Figure 5). There is a small difference between soybean and pea, and this may be attributed to different volumes occupied by lipid that we do not expect to be responsive to added moisture; lipid content for soybean and pea embryonic axes are 0.155 + 0.004 and 0.097 + 0.002 g lipid g^–1^ dw, respectively. At 25°C, 0.1 g g^–1^ corresponds to between 48 and 55% RH for pea and soybean embryonic axes, respectively (data not shown).

Fluctuation between fluid and solid states, so above and below about 50–60% RH at about 20–30°C, will have profound effects on physiological change. Properties at moisture contents slightly above Tg are governed by glass fragility, a central property of glasses that dictates viscosity with small changes in temperature and moisture. In this paper, we show that pea seeds survive longer over a broader moisture range above the α relaxation (vertical line in Figure 1) compared to soybean seeds, suggesting that the axes of pea have less fragility. Fragility is more directly measured by changes in viscosity through Tg [Walters, 2004; Ballesteros et al., 2018 (and references within)]. Here, we believe that the slopes of the α relaxation (Table 2) and the values of Δ tan δ in response to moisture are indicative of packing efficiency and so probably co-correlated with fragility (Kunal et al., 2008). Seed longevity has been reported for seeds stored in uncontrolled conditions where RH fluctuates above Tg (Priestley, 1986). Longevity is also predicted by models that more-or-less describe the solidification process but not solid *per se* [e.g., the Viability Equations (Ellis and Roberts, 1980; Sun, 1997; Hay et al., 2003)]. We found poor correlations between these measures of longevity and those of seeds within the genebank, that assuredly have solidified cytoplasm (Walters et al., 2005a,b; Buitink and Leprince, 2008), possibly because of the differences in fragility among diverse seeds. Therefore, it would be interesting to correlate DMA indicators of fragility or plasticization efficiency with a measured moisture coefficient (C_w_, Royal Botanic Gardens Kew, 2019) used to predict longevity in empirically based models.

Fragility within a seed can be regulated locally by differences in space-filling solutes or flexibility of side chains of polymers (Dudowicz et al., 2008; Kunal et al., 2008; Stukalin et al., 2010). We detected differences in tissue responses to moisture in pea axes compared to cotyledons, with the axes having properties reflecting greater fragility based on the slopes of α relaxation temperature to moisture (Table 2) and values of Δ tan δ (Figure 8). Differences in glass fragility among tissues and even within cells provides a material science-based explanation for localized differences in metabolism and fluidity within an anhydrobiote (Leubner-Metzger, 2005; Choi et al., 2011).

Glass strength and stability correlate with the separation of α and β relaxations, presumably because wider separation de-couples long and short-range motions that affect potential for inter-molecular interaction contributing to degradation (Perez et al., 1999; Poirier-Brulez et al., 2006; Yoshioka and Aso, 2007; Yu et al., 2013; Micoulaut, 2016). Close to the solid ↔ fluid transition, α and β relaxations are practically merged (Figure 6, β relaxation peaks (open triangles) appear within the region of α relaxation onset and peak; Figure 7A, T_α_ − T_β_ = 0 at about 0.09 g H_2_O g^–1^ dm). These relaxation events become separated with further drying of the solid (Figures 3, 7A) and are a possible explanation for the increasing longevity of both pea and soybean as they are dried below Tg (Figure 1). In pea axes near about 0.06 g H_2_O g^–1^ dw, the trend toward greater separation of α and β relaxations changes slope (Figure 7A), and at this point, P50 also begins to decline with further drying (Figure 1). The same break is not observed in the T_α_ − T_β_ function in soybean axes, but occurs in the T_β_ − T_γ_ function, where these relaxations actually come closer together with further drying (Figure 7B). Limits to the beneficial effect of drying on longevity at a critical water content near 18–25% RH (at the storage temperature) have been acknowledged for years (Ellis et al., 1990; Vertucci and Roos, 1990; Walters et al., 2005a; Ballesteros et al., 2018). Perhaps greater coupling of molecular motions, which we detect as changed separation of relaxations events, provides a plausible physical explanation.

The size of tan δ (Δ tan δ) at the α relaxation is, perhaps, the most observable difference between mechanical properties of pea and soybean axes. The size of α relaxations reflects molecular participation in the relaxation event (Priestley et al., 2005), and the larger Δtan δ in soybean compared to pea axes (Figures 3C, 8A) suggests there are less molecular restrictions and greater possibility for diffusional movement, probably due to lower packing density or higher flexibility of molecules. Decreasing water content also decreases Δtan δ for α and β relaxations in pea and β relaxations in soybean (Figures 3, 4, 7), indicating that the compressive force used here in DMA experiments was insufficient to effect deformation in severely dried cells, especially pea, again pointing to high rigidity within this cytoplasm.

The absolute value of either the storage (E′) or loss (E^″^) modulus at a particular moisture-temperature combination is informative about the rigidity or viscosity within the matrix; however, high variation among replicate samples – probably related to geometry and minor differences of probe contact on the material – precluded confident quantification of point values for moduli. A surrogate measure that eliminates some of the point level variability may be the slope of the E′ relationship with temperature ($\frac{\partial E^{\prime }}{\partial T}$, Figure 3A) which indicates the sensitivity of the solid matrix to temperature, independent of moisture. DMA scans of extremely dry pea axes (Figure 3A) and cotyledons (Ballesteros and Walters, 2011) were fairly shallow, but became increasingly steep when moisture was added (negative slope in Table 1). In contrast, the solid matrix in very dry soybean axes showed high sensitivity to temperature (Figure 4A, compare intercepts in Table 1), and addition of water had some effect on $\frac{\partial E^{\prime }}{\partial T}$, but not to the extent observed in pea (compare slopes in Table 1). These observations are an additional way of quantifying the rigidity of structure in pea compared to soybean, again suggesting that the solid matrix in soybean allows more diffusive motion and is more fragile (*sensu* Angell) than pea.

Mechanical properties of solid matrices within pea and soybean axes were quantified as separation of relaxations (T_α_ – T_β_ and T_β_ – T_γ_, Figure 5B), the size of the tan δ function at the α relaxation (Δ tan δ, Figure 2C), and the changed slope of the storage modulus ($\frac{\partial E^{\prime }}{\partial T}$, Figure 3A) with addition of a plasticizer, such as water. These reveal insights about core thermal properties of these glasses, namely Tg and fragility, and suggest greater stability within the cytoplasm of pea axes which is fairly resilient to change by increasing temperature and moisture, while small changes in these conditions can result in large changes in structure and mobility of molecules within the cytoplasm in dried soybean axes. The greater susceptibility for increased relaxation within the glassy matrix of soybean suggests that it will be more prone to physical aging, that is, the slow compression of the glassy matrix with time (Struik, 1977). A collection of soybean and pea cohorts dating back to the 1990s is available to compare changed mechanical properties in fresh and deteriorated seeds using DMA as well as hypothesized increased viscosity using detailed heat capacity measurements and principles of configureurational entropy (Walters, 2004).

## Conclusion

Mechanical properties are frequently used to predict material performance and how performance might change with different composition, processing methods, or environmental conditions. The measurements reported in this paper seek to characterize mechanical properties within dry cytoplasm of pea and soybean embryonic axes to provide insight about their respective long and short life spans as well as reduced longevity with extreme drying. Solid matrices within soybean axes show evidence of high long-range mobility, are easily plasticized by water and reflect properties consistent with fragile glasses, suggesting that they may be prone to physical aging. In contrast, structures within pea axes have low mobility over broad ranges of moisture and temperature – perhaps due to steric hindrances at macromolecular surfaces. We clearly show that solid-state properties among tissues in seeds can vary and we point out that localized differences in glass fragility (*sensu* Angell), usually controlled by ligand flexibility, can lead to variation in glassy structure – from highly viscous to completely fluid – in different tissue types of regions of the cell that are stored under identical moisture and temperature conditions.

## Data Availability

All datasets generated for this study are included in the manuscript and/or the Supplementary Files.

## Author Contributions

CW conceived the project and wrote the proposal funding DB’s fellowship. DB designed and performed the experiments and analyzed the data. DB and CW wrote the manuscript and contributed to the revisions.

## Disclaimer

Mention of trade names or commercial products in this article is solely for the purpose of providing specific information and does not imply recommendation or endorsement by the U.S. Department of Agriculture. USDA is an equal opportunity provider and employer.

## Conflict of Interest Statement

The authors declare that the research was conducted in the absence of any commercial or financial relationships that could be construed as a potential conflict of interest.
