# Supplementary figures and images for: Solid-State Biology and Seed Longevity: A Mechanical Analysis of Glasses in Pea and Soybean Embryonic Axes
Source: Front Plant Sci. 2019 Jul 16;10:920. doi: 10.3389/fpls.2019.00920 (PMC6646689; doi:10.3389/fpls.2019.00920)

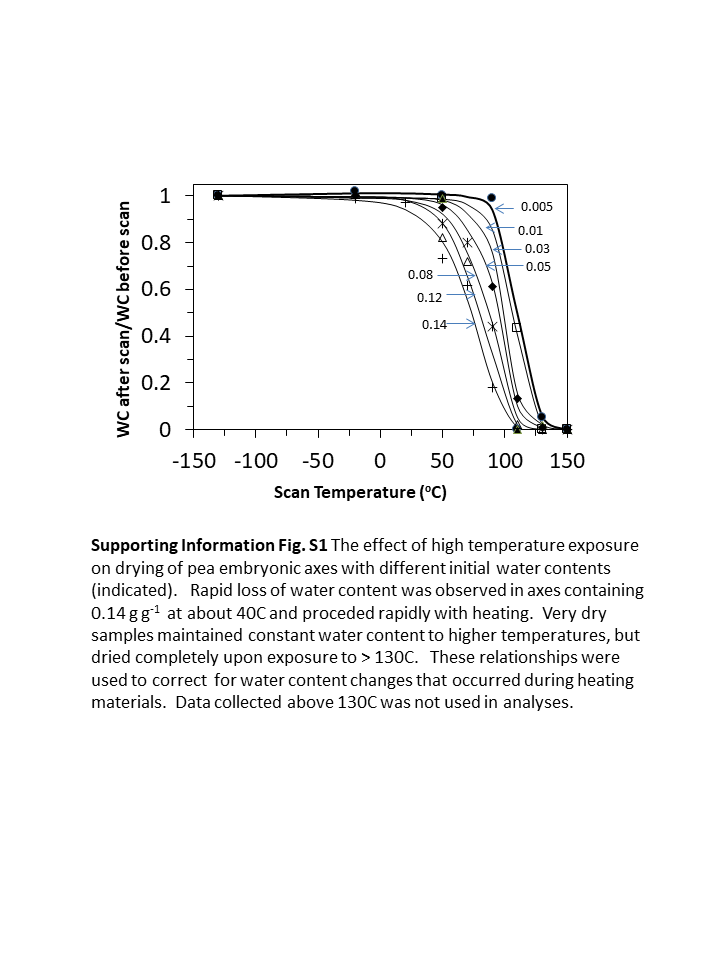

Supplement: Supplementary file 1 [file Image_1.TIF]
